# Interhemispheric asymmetry of c‐Fos expression in glomeruli and the olfactory tubercle following repeated odor stimulation

**DOI:** 10.1002/2211-5463.12851

**Published:** 2020-04-13

**Authors:** YoonGyu Jae, NaHye Lee, Dae Won Moon, JaeHyung Koo

**Affiliations:** ^1^ Department of Brain and Cognitive Sciences DGIST Daegu Korea; ^2^ Department of New Biology DGIST Daegu Korea; ^3^ Center for Bio‐Convergence Spin System DGIST Daegu Korea

**Keywords:** AONpE, asymmetry, c‐Fos, glomeruli, olfactory tubercle

## Abstract

Odor adaptation allows the olfactory system to regulate sensitivity to different stimulus intensities, which is essential for preventing saturation of the cell‐transducing machinery and maintaining high sensitivity to persistent and repetitive odor stimuli. Although many studies have investigated the structure and mechanisms of the mammalian olfactory system that responds to chemical sensation, few studies have considered differences in neuronal activation that depend on the manner in which the olfactory system is exposed to odorants, or examined activity patterns of olfactory‐related regions in the brain under different odor exposure conditions. To address these questions, we designed three different odor exposure conditions that mimicked diverse odor environments and analyzed c‐Fos‐expressing cells (c‐Fos+ cells) in the odor columns of the olfactory bulb (OB). We then measured differences in the proportions of c‐Fos‐expressing cell types depending on the odor exposure condition. Surprisingly, under the specific odor condition in which the olfactory system was repeatedly exposed to the odorant for 1 min at 5‐min intervals, one of the lateral odor columns and the ipsilateral hemisphere of the olfactory tubercle had more c‐Fos+ cells than the other three odor columns and the contralateral hemisphere of the olfactory tubercle. However, this interhemispheric asymmetry of c‐Fos expression was not observed in the anterior piriform cortex. To confirm whether the anterior olfactory nucleus pars externa (AONpE), which connects the left and right OB, contributes to this asymmetry, AONpE‐lesioned mice were analyzed under the specific odor exposure condition. Asymmetric c‐Fos expression was not observed in the OB or the olfactory tubercle. These data indicate that the c‐Fos expression patterns of the olfactory‐related regions in the brain are influenced by the odor exposure condition and that asymmetric c‐Fos expression in these regions was observed under a specific odor exposure condition due to synaptic linkage via the AONpE.

## Graphical abstract

Odor adaptation is essential for the olfactory system to maintain high sensitivity to persistent and repetitive odor stimuli. Here, we examine the differences in the proportions of c‐Fos‐expressing cells depending on the odor exposure conditions. Asymmetric c‐Fos expression in glomerulus and the olfactory tubercle were observed under repeated odor stimulation due to synaptic linkage via the anterior olfactory nucleus pars externa.

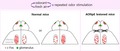

**Abbreviations**
AONpE: anterior olfactory nucleus pars externa
*C. elegans*: *Caenorhabditis elegans*
c‐Fos+ cells: c‐Fos‐expressing cells
continuous: the continuous condition
GFP+ odor column: green fluorescent protein‐expressing column
IBA: ibotenic acid
IL: intensively c‐Fos‐labeled lateral
IRES: internal ribosome entry site
JG cells: juxtaglomerular cells
LL: left lateral
LM: left medial
MOR23 glomeruli: MOR23‐targeted glomeruli
multi: the multiple pulsed condition
OB: olfactory bulb
odor column: the MOR23 glomerulus and its surrounding cells
OR: olfactory receptor
OSN: olfactory sensory neuron
PBST: Triton X‐100 in phosphate‐buffered saline
RL: right lateral
RM: right medial
single: the single pulsed condition
TH: tyrosine hydroxylase

Olfactory sensory neurons (OSNs) in the epithelium expressing a given type of olfactory receptor (OR) project their axons to glomeruli in each OB [1, 2, 3]. Four isogenetic glomeruli are symmetrically distributed over the left and right OBs [4, 5, 6]. A pair of glomeruli on the same side of the OB form an intrabulbar inhibitory network through tufted cells, and the interbulbar inhibitory network is constructed between two lateral glomeruli on each side of the OBs by external tufted cells and the anterior olfactory nucleus pars externa (AONpE) [7, 8, 9, 10]. Juxtaglomerular (JG) cells, which surround the glomeruli, modulate neural activation from OSNs, communicate with other glomeruli and transfer information from the higher brain [11]. The information modified within the glomerulus is transmitted through the mitral and tufted cells to the piriform cortex and the olfactory tubercle to complete olfactory cognition.

Odor adaptation refers to the ability of the olfactory system to regulate sensitivity at different stimulus intensities, which is essential for preventing saturation of the cell‐transducing machinery and maintaining high sensitivity to persistent and repetitive odor stimuli [12]. The time dependence of initiating odor adaptation and recovery plays a major role in determining the temporal response characteristics of the olfactory system during both processes [13]. Previous studies have mainly focused on one of the activities of OSNs, glomeruli, and its related cells or cortex by controlling the exposure time to odorants, and have characterized the physiological roles of channels or secondary ion messengers in OSNs that respond to odorants [12, 14, 15, 16, 17, 18]. However, few studies have considered differences in neuronal activation depending on the diverse ways of inputting odors to the OB and olfactory cortex, and studies on the precise understanding of odorant exposure conditions and the activity patterns of olfactory‐related regions in the brain are needed.

To address these issues, we designed a diverse odor exposure environment for mice and analyzed neural activity patterns in the odor columns and the olfactory cortex. We chose experimental areas where the neural signal is transmitted by the OSNs. Thus, we selected the JG cells surrounding the glomeruli, the olfactory tubercle, and the piriform cortex as the experimental target regions. We then evaluated quantitative patterns of c‐Fos expression and the proportion of c‐Fos‐expressing cell types in the three brain olfactory regions by combining genetically identified OR transgenic mice under three different odorant exposure conditions that mimicked the olfactory environment and used c‐Fos and cellular marker immunolabeling techniques to detect the responses. We concluded that the c‐Fos expression patterns of the olfactory‐related regions in the brain are influenced by diverse ways of odor exposure, and asymmetry of c‐Fos expression in the brain was observed under a specific odor exposure condition.

## Results

**Fig. 1:**
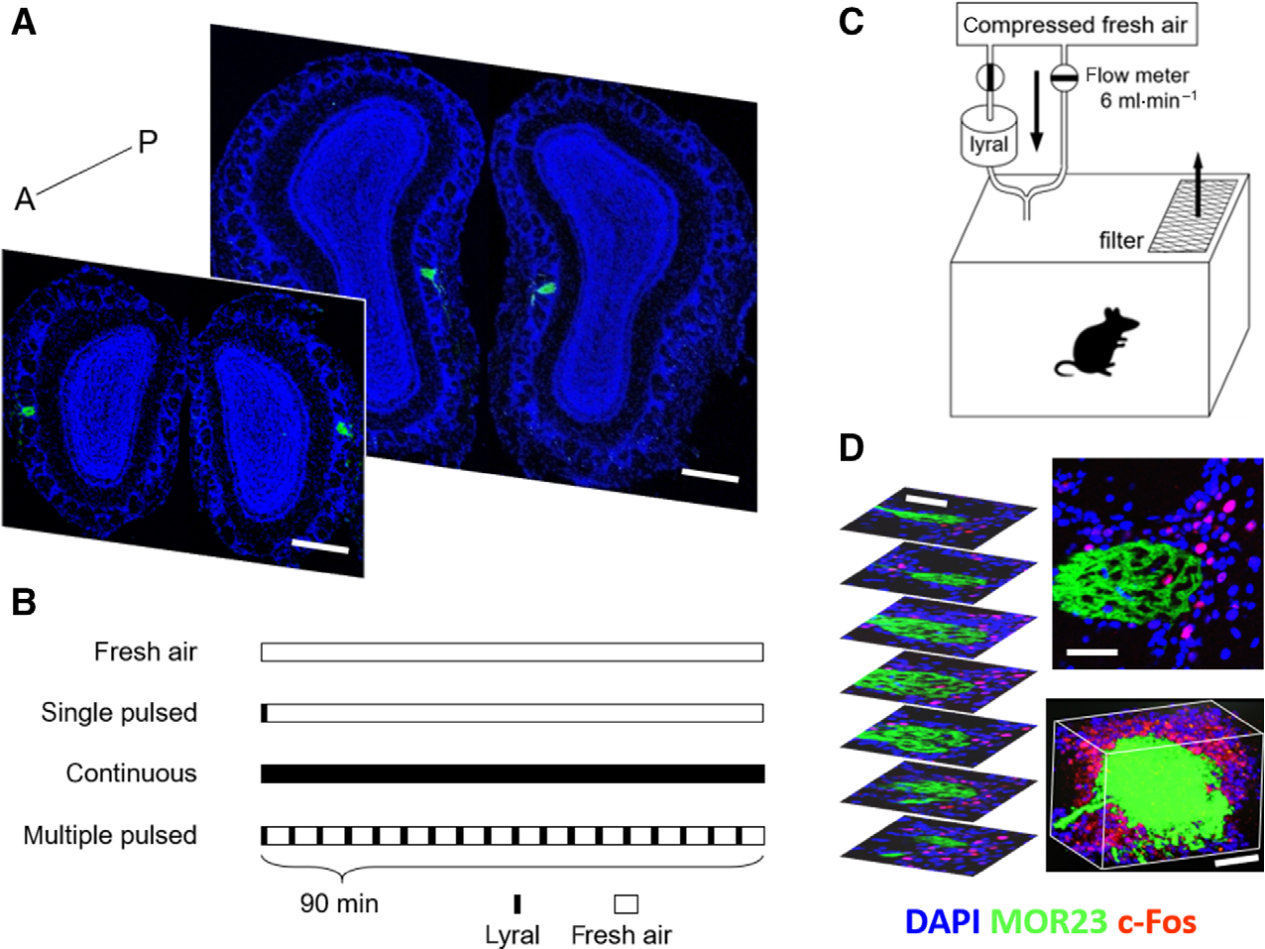
Localization of MOR23 glomeruli and experimental design. (A) Two pairs of GPF‐expressing MOR23 glomeruli in OB. One pair located anterolateral and the other pair located posteromedial in the OB. A, anterior; P, posterior. Scale bar, 300 µm. (B) Three different odor exposure conditions with a negative control. Black rectangles, air flow with lyral; white rectangles, fresh air flow. In the single pulsed and multiple pulsed conditions, duration of lyral exposure time was 1 min. (C) Odorant exposure chamber. Highly purified compressed fresh air was delivered to chamber with or without lyral. Application of lyral was controlled with two independent air flow meters. (D) c‐Fos immunostaining of the odor columns and 3D reconstruction. Left panel, serial sections of the complete structure of the MOR23 odor columns; right upper panel, representative image of c‐Fos‐labeled MOR23 odor column; right bottom panel, captured image of a 3D reconstructed movie clip. Scale bar, 50 µm.

We utilized MOR23‐internal ribosome entry site (IRES)‐tauGFP mice and lyral (Fig. S1), an odorant that stimulates MOR23 [19, 20]. In these mice, the MOR23 targeted glomeruli (MOR23 glomeruli) can be identified by GFP expression. We analyzed 1985 images from 78 mice. In our morphological analysis, most of the mice had two exact pairs of MOR23 glomeruli (~ bregma 4.63 mm and 4.10 mm), except three mice (Fig. 1A). To investigate neural activation of the OB after exposure to the odorant, we examined the induction of c‐Fos as a marker of neural activation of JG cells (Fig. 1D, right upper panel). The duration of the odor exposure time was 90 min according to the results of Kimoto *et al*. (2005) (see Discussion). The odorant was applied in three different ways (Fig. 1B):

1. Exposing the odorant for the first min followed by fresh air for the next 89 min [referred to as the single pulsed condition (single)]
2. Continuous exposure to the odorant for 90 min (referred to as the continuous condition [continuous])
3. Repeated exposure to the odorant for 1 min at 5‐min intervals over a 90‐min period [referred to as the multiple pulsed condition (multi)]

A mouse was placed in a custom‐made odorant stimulation chamber (Fig. 1C). The odorant was applied by two divided air flow meters. A series of OB tissue sections were c‐Fos stained to elucidate complete neuronal activation of the JG cells around the MOR23 glomeruli (Fig. 1D, left panel). 3D movie clips of the glomeruli and its JG cells were made from multi by z‐stack reconstructing 70–100 images (Fig. 1D, right bottom panel, https://doi.org/10.6084/m9.figshare.11602155.v1, Video S1–S4). Henceforth, the MOR23 glomerulus and its surrounding cells were referred to as an odor column [8, 10, 21, 22]. 

**Fig. 2:**
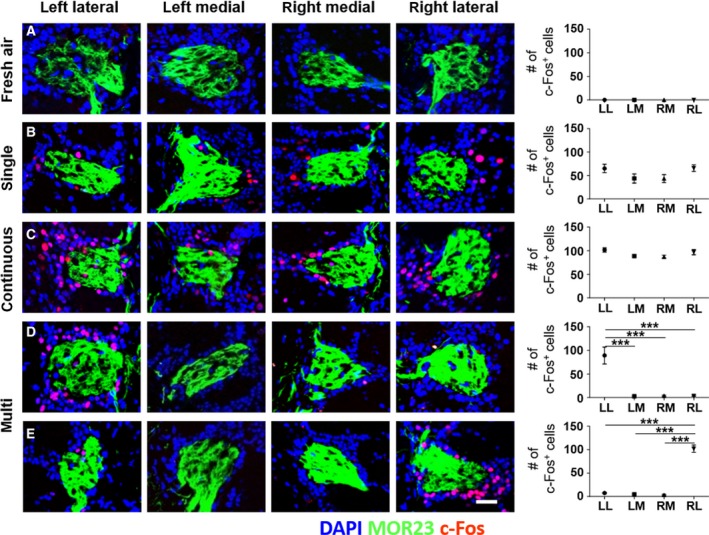
Distribution of c‐Fos expression in four odor columns depending on the three different odor exposure conditions. (A) c‐Fos expression in the odor columns by exposure of odorless fresh air as the negative control. (B, C) Representative images of c‐Fos expression in odor columns under the single pulsed and continuous. All four odor columns expressed c‐Fos and no difference was detected between the left and right side of the OB. More c‐Fos‐immunoreacted cells were detected under the continuous than the single in both lateral and medial columns. (D, E) Two representative patterns of c‐Fos expression under multi. One of the lateral odor columns exhibited intensive c‐Fos expression but the other lateral and pair of medial odor columns poorly immunoreacted. Compared with the total number of analyzed mice in (D) and (E), no predominant side was observed between left and RL columns (D = 11 mice; E = 13 mice). Data are mean ± SEM (*n* = 5 for A, *n* = 24 for B–E). ***P < 0.001 by one‐way ANOVA with Tukey’s *post hoc* correction. Fresh air, fresh air exposure condition; continuous, the continuous. Scale bar, 50 µm.

To elucidate neural activation of the odor columns in response to the three different odorant exposure conditions, c‐Fos labeling and a quantitative analysis were conducted with two pairs of the entire column structure (Fig. 2). The mice were exposed to odorless fresh air as the negative control; we rarely found c‐Fos‐expressing cells (c‐Fos+ cells) in this condition (Fig. 2A). Fewer c‐Fos‐immunoreacted cells were detected in the odor columns under the single (Fig. 2B) compared with the continuous (Fig. 2C). No significant difference in c‐Fos expression was detected between the left and right odor columns for the single pulsed and continuous. Surprisingly, one of the lateral odor columns was abundantly labeled with c‐Fos under multi (referred to as an intensively c‐Fos‐labeled column), whereas the other three odor columns rarely presented c‐Fos labeling (Fig. 2D,E). Among the 24 mice analyzed, 11 presented with intensive c‐Fos labeling in the left lateral (LL) column (Fig. 2D) and 13 expressed c‐Fos in the right lateral (RL) column (Fig. 2E).

**Fig. 3:**
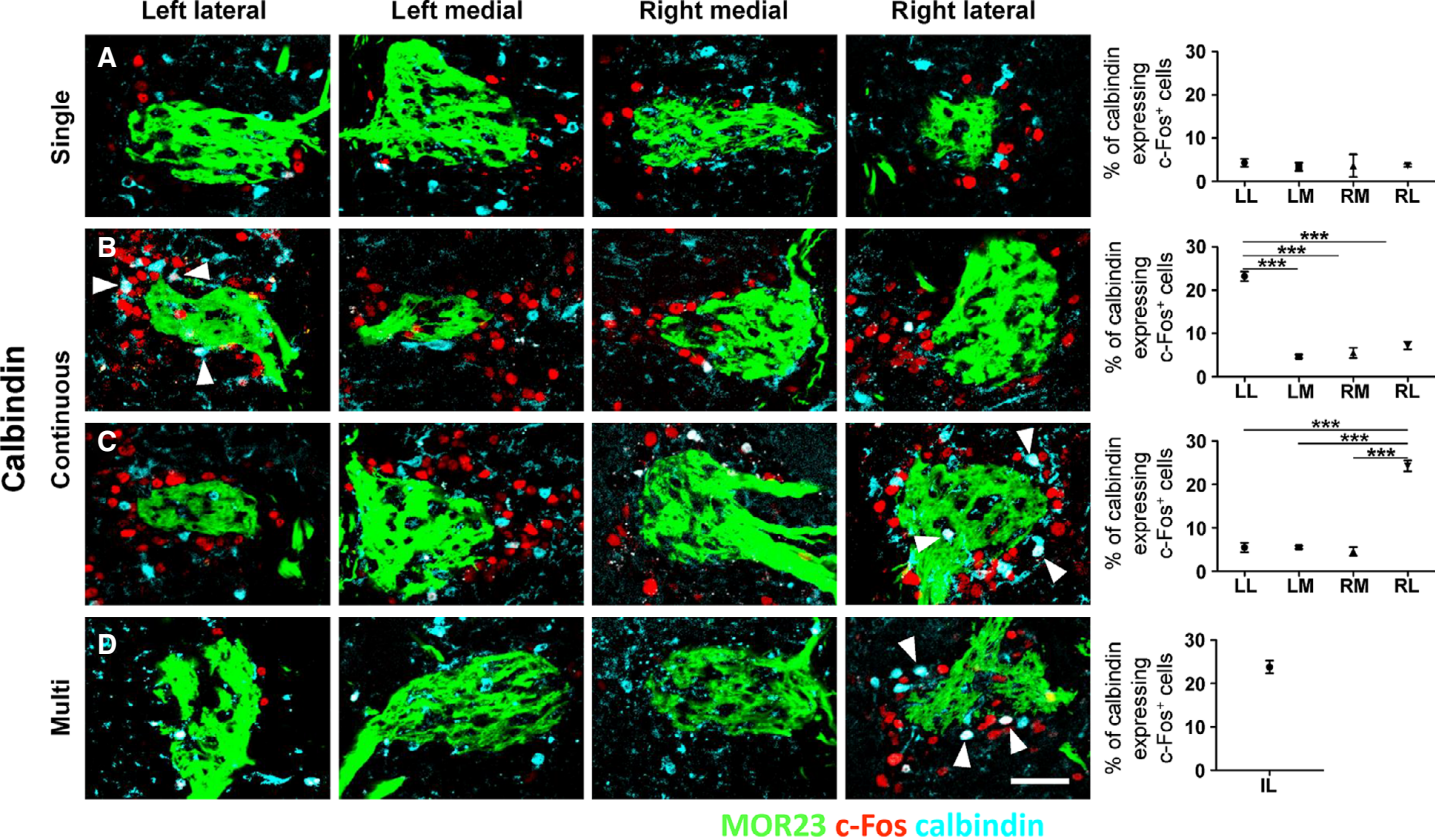
Distribution of calbindin‐expressing c‐Fos^+^ cells in the four odor columns depending on the odor exposure condition by co‐immunolabeling analysis (A) In the single, the proportion of calbindin‐expressing c‐Fos^+^ cells was not different between the two pairs of odor columns. (B, C) In the continuous, two different asymmetric proportions of calbindin‐expressing c‐Fos^+^ cells were observed. One of the lateral columns presented an obviously higher proportion of calbindin‐expressing c‐Fos^+^ cells (arrowheads, white colored cells) and the other lateral and two medial columns showed same proportion compared with columns in (A). No side was predominant between the LL and RL columns (B = 6 mice; C = 7 mice). (D) In multi, the proportion of calbindin‐expressing c‐Fos^+^ cells (arrowheads, white colored cells) in an intensively c‐Fos‐labeled column was not significantly different with the LL column of (B) and the RL column of (C). Data are mean ± SEM (*n* = 5–7 for A–D). ***P < 0.001 by one‐way ANOVA with Tukey’s *post hoc* correction. Scale bar, 50 µm.

**Fig. 4:**
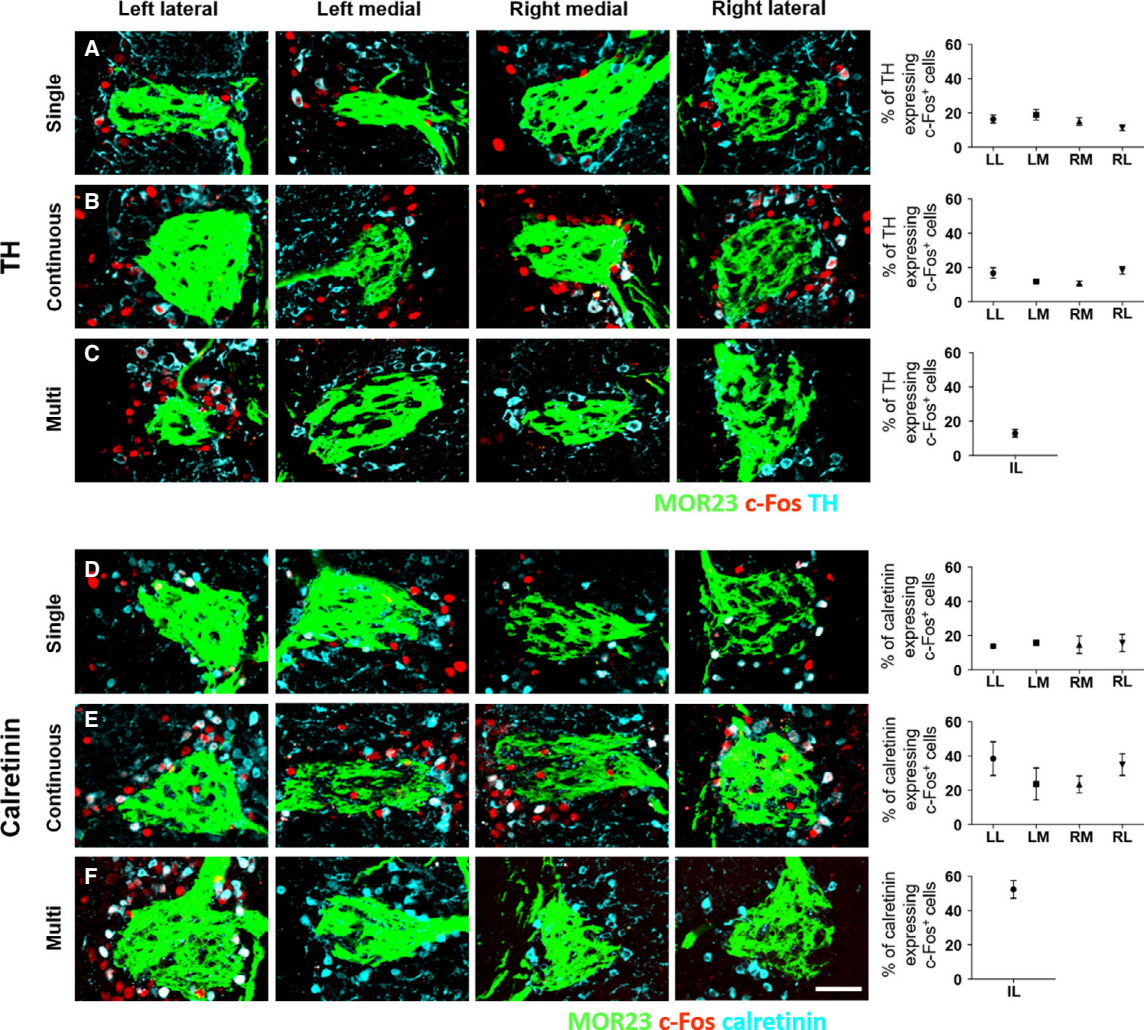
Distribution of TH‐expressing c‐Fos^+^ cells (A–C) and calretinin‐expressing c‐Fos^+^ cells (D–F) in the four odor columns depending on the odor exposure condition by co‐immunolabeling analysis. No asymmetric c‐Fos expression was observed between the left and right OBs. (A, B) In the single pulsed and continuous, the proportion of TH‐expressing c‐Fos^+^ cells in the four odor columns was not affected by the odor exposure condition. (C) In multi, the proportion of TH‐expressing c‐Fos^+^ cells in an intensively c‐Fos‐labeled column was not significantly different from the columns in (A) and (B). (D, E) In the single pulsed and continuous, the proportion of calretinin‐expressing c‐Fos^+^ cells in four odor columns was not different from each other. Compared with (D) and (E), the number of calretinin‐expressing c‐Fos^+^ cells under the continuous was more than the number of calretinin‐expressing c‐Fos^+^ cells under the single. (F) In multi, the proportion of calretinin‐expressed c‐Fos^+^ cells in an intensively c‐Fos‐labeled column was not significantly different with the columns in (E). Data are as mean ± SEM (*n* = 5–8 for A–F). Scale bar, 50 µm.

The next analysis was a cell‐type distribution of c‐Fos‐labeled neurons under the three different odor exposure conditions, so we colabeled the odor columns with c‐Fos and a JG cell marker (Figs 3,4 and Table S1–S3). The JG cells surrounding the glomerulus in the odor column are divided into three largest populations, each consisting of calbindin, tyrosine hydroxylase (TH), and calretinin‐expressing cells [11]. In addition, each cell type is known to have different synaptic properties of the odor column [23, 24, 25]. Therefore, we hypothesized that analyzing cell‐type distribution of c‐Fos^+^ cells contributes to the asymmetric pattern depending on odor exposure conditions. We utilized three markers, such as calbindin, TH, and calretinin, to characterize c‐Fos^+^ cells [11, 26]. Before proceeding with this experiment, we conducted a confirmatory experiment to check whether the three markers labeled different cells (Fig. S3). As a result, the three markers labeled different cells in the OB, including those near the MOR23 column area.

No difference in the number of calbindin‐expressing c‐Fos^+^ cells was observed in the four columns under the single (Fig. 3A). Strikingly, only one of the lateral columns among four c‐Fos‐labeled columns presented a higher proportion of calbindin‐expressing c‐Fos^+^ cells compared with the other three columns under the continuous (Fig. 3B,C, arrowheads). No side was predominant among the 13 analyzed mice. In addition, the proportion of calbindin‐expressing c‐Fos^+^ cells in an intensively c‐Fos‐labeled column under multi was not significantly different from the proportion of a higher calbindin‐ and c‐Fos‐labeled column under the continuous (Fig. 3B, left panel; Fig. 3C, right panel; Fig. 3D, right panel, arrowheads). Taken together, the asymmetric distribution of calbindin‐expressing c‐Fos^+^ cells was observed between the LL and RL odor columns especially under the continuous.

Next, we analyzed the proportion of TH‐expressing c‐Fos^+^ cells under the three different odor exposure conditions (Fig. 4A–C). The four odor columns contained statistically same proportions of TH‐expressing c‐Fos^+^ cells under the single pulsed and continuous (Fig. 4A,B). In multi, the proportion of TH‐expressing c‐Fos^+^ cells in an intensively c‐Fos‐labeled column was not significantly different from the columns in the single pulsed and continuous (Fig. 4C). Taken together, the proportion of TH‐expressing c‐Fos^+^ cells in the four odor columns was not affected by the differences in the odorant exposure conditions.

The proportion of calretinin‐expressing c‐Fos^+^ cells was also analyzed under the three different odor exposure conditions (Fig. 4D–F). The proportion of calretinin‐expressing c‐Fos^+^ cells under the continuous was higher than the proportion of calretinin‐expressing c‐Fos^+^ cells under the single in all four columns (Fig. 4D,E. *P* < 0.05 by unpaired Student’s *t*‐test. LL, *P* = 0.0008; left medial (LM), *P* < 0.0001; right medial (RM), *P* = 0.007; RL, *P* = 0.0121). These results indicate that the c‐Fos^+^ cells are more abundant under the continuous than under the single in the four odor columns (Fig. 2B,C), presumably due to the increased number of calretinin‐expressing c‐Fos^+^ cells. In multi, the proportion of calretinin‐expressing c‐Fos^+^ cells in an intensively c‐Fos‐labeled column was not significantly different from the lateral columns under the continuous (Fig. 4F). Additionally, the asymmetric distribution of c‐Fos^+^ cells that expressed TH (Fig. 4A,B) or calretinin (Fig. 4D,E) was not observed between the left and right OBs.

**Fig. 5:**
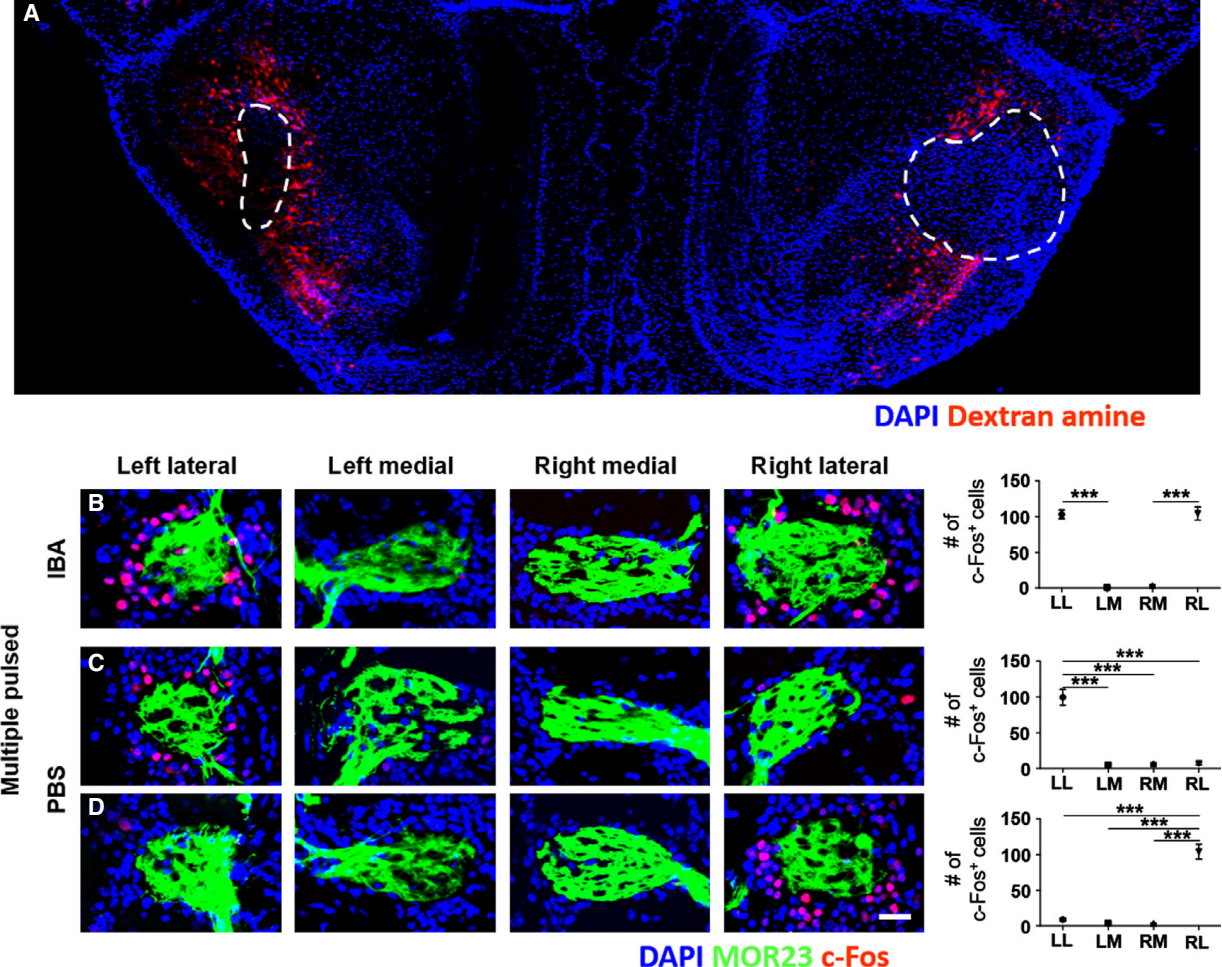
Contribution of the AONpE to asymmetric c‐Fos expression in a pair of lateral odor columns under multi. (A) Injection site of IBA over the AONpE region (dashed line). Scale bar, 0.5 mm. (B) Symmetric c‐Fos immunoreactivity was observed in the two lateral odor columns by the lesions in the AONpE under the multiple pulsed odor stimulation. (C, D) Negative control. Asymmetric c‐Fos expression was observed in the lateral odor columns of the PBS‐injected mice under multi. Scale bar, 50 µm. Data are mean ± SEM (*n* = 5 for B, *n* = 13 for C, D). ***P < 0.001 by one‐way ANOVA with Tukey’s *post hoc* correction. IBA, IBA‐injected mice; PBS, PBS‐injected mice.

The interhemispheric odor columns communicate with each other using a well‐characterized synaptic structure, particularly between lateral columns via the AONpE, which is an inhibitory linkage of granule cells [9, 10, 27, 28]. We assumed that the lateral odor columns suppressed expression of c‐Fos in the contralateral odor column via the AONpE under multi (Fig. 5 and Table S4). To test whether the AONpE is necessary for asymmetric c‐Fos expression under multi, we lesioned both sides of the AONpE by pressure injecting ibotenic acid (IBA), which lesioned the soma of the neurons in the injected areas (Figs 5A and S4). Then, we examined the c‐Fos expression patterns in the odor columns following multi after injecting IBA or PBS. Symmetric c‐Fos expression was observed in the two lateral odor columns of the bilateral‐lesioned mice, but we rarely detected c‐Fos^+^ cells in the two medial odor columns (Fig. 5B). Asymmetric c‐Fos expression patterns were observed in the PBS‐injected mice (Fig. 5C,D). Thus, we concluded that asymmetric c‐Fos expression patterns in the two lateral odor columns may be regulated by the interhemispheric inhibitory network via the AONpE. We also tested single‐side‐lesioned mice (lesion for left side of the AONpE only or right side of the AONpE only) under multi but rarely detected any particular symmetric or asymmetric distribution of c‐Fos expression in the two pairs of odor columns (Table S5). Unilateral PBS‐injected mice under multi exhibited asymmetric c‐Fos expression in lateral odor columns as in the bilateral‐lesioned mice.

**Fig. 6:**
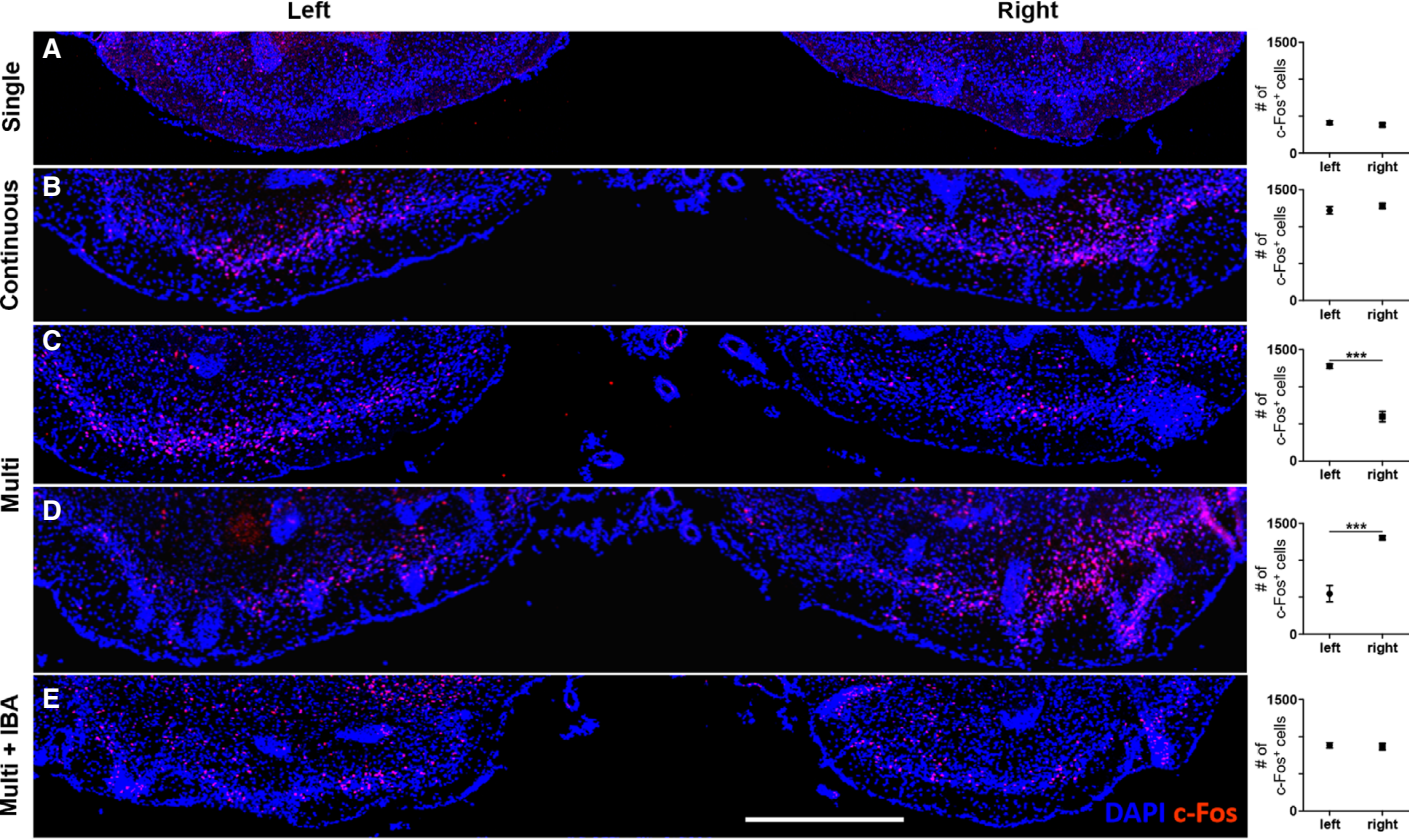
Distribution of c‐Fos^+^ cells in the olfactory tubercle depending on the three different odor exposure conditions. Representative images from the whole olfactory tubercle were selected in the same region (bregma 1.21 mm to bregma 0.97 mm). (A, B) Asymmetric c‐Fos immunoreactivity was rarely observed in the left and right olfactory tubercles under the single pulsed or continuous. A quantitative analysis indicated that the number of c‐Fos^+^ cells under the continuous was more than the number of c‐Fos^+^ cells under the single. (C, D) Asymmetric c‐Fos expression was observed in the left or right hemispheres of the olfactory tubercle under multi. Complete correlation with two lateral odor columns in the OB compared with Fig. 2D,E. (C = 11 mice; D = 13 mice). (E) No asymmetry of c‐Fos expression was observed under multi with the AONpE‐lesioned mice. c‐Fos immunoreactivity was quantitatively reduced in both the left and right hemispheres of the olfactory tubercles compared to (B), left hemisphere of the olfactory tubercle in (C), and right hemisphere of the olfactory tubercle in (D), respectively. Data are mean ± SEM (*n* = 5 for A, B, and E; *n* = 24 for C, D). ***P < 0.001 by unpaired Student’s *t*‐test. multi + IBA, IBA‐injected mice under multi. Scale bar, 0.5 mm.

**Fig. 7:**
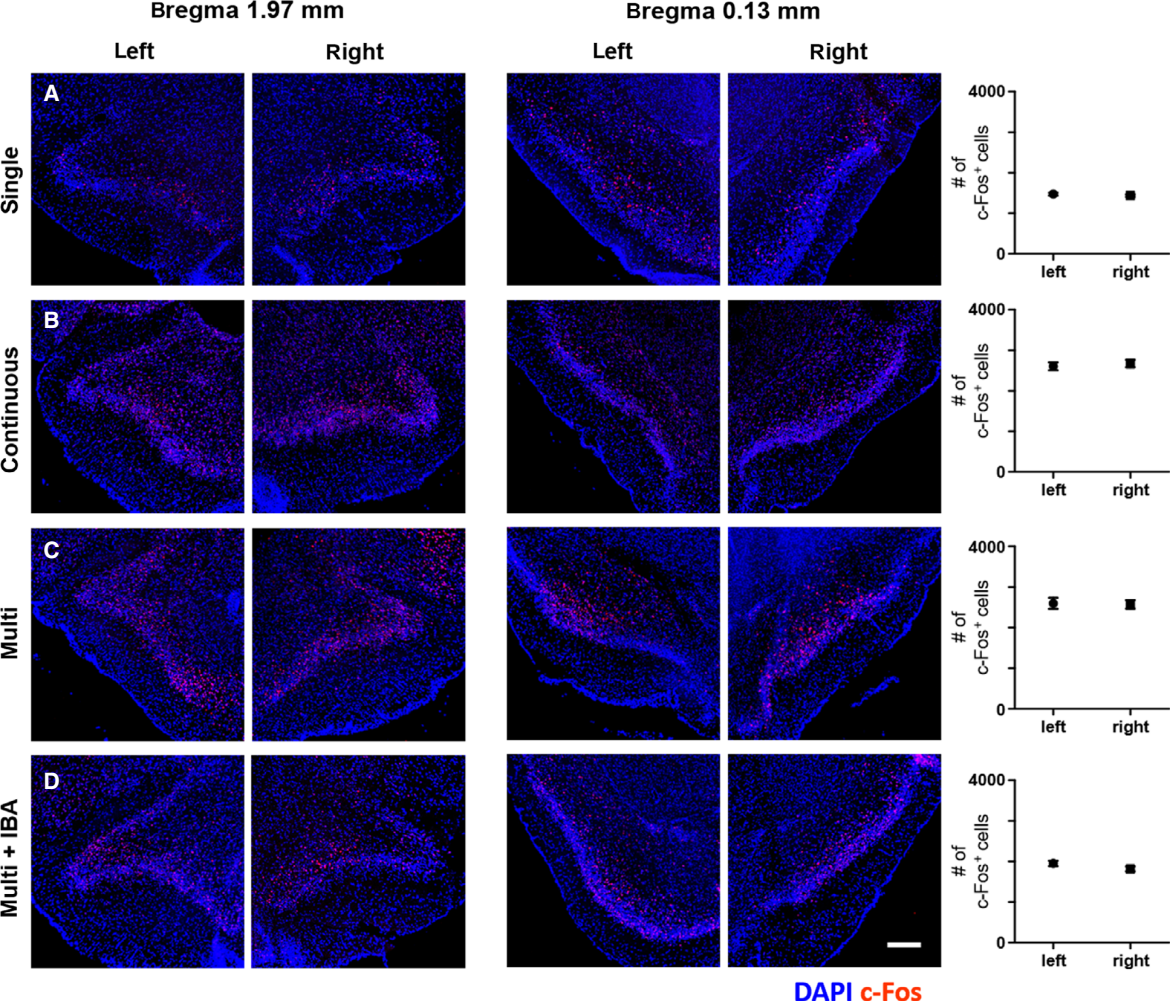
Distribution of c‐Fos^+^ cells in the anterior piriform cortex depending on the three different odor exposure conditions. (A–D) Asymmetric c‐Fos expression was rarely observed in the anterior piriform cortex under any of the odor exposure conditions. Representative images were selected from two regions, bregma 1.97 mm (left panel) and bregma 0.13 mm (right panel) respectively. (A) In the single, the lowest c‐Fos expression was observed in the anterior piriform cortex compared to the other odor exposure conditions. (B, C) Abundant c‐Fos expression was observed under the continuous and multiple pulsed conditions in the anterior piriform cortex. (D) Reduced c‐Fos expression was observed under multi in the AONpE‐lesioned mice compared with (C). Data are mean ± SEM (*n* = 5–7 for A–D). Range of no significance is > 0.05 by unpaired Student’s *t*‐test. multi + IBA, IBA‐injected mice under multi. Scale bar, 0.2 mm.

To further test whether c‐Fos expression in the two pairs of odor columns was also correlated in the higher olfactory cortex, we characterized c‐Fos expression in the olfactory tubercle and piriform cortex depending on the odor exposure conditions (Figs 6,7, and Tables S6–S7). We rarely found c‐Fos^+^ cells in both hemispheres of the olfactory tubercles under the odorless fresh air exposure condition as the negative control (data not shown). Significantly more cells were c‐Fos labeled in both hemispheres of the olfactory tubercles under the continuous than the single (Fig. 6A,B). We rarely detected any asymmetric distribution of c‐Fos expression in the left and right hemispheric olfactory tubercles under the single pulsed or continuous. Strikingly, following multi, significantly more cells expressed c‐Fos in one hemisphere of the olfactory tubercle compared with another hemisphere of the olfactory tubercle (Fig. 6C,D). This left/right asymmetry of c‐Fos expression in the olfactory tubercle (11 mice for Fig. 6C; 13 mice for Fig. 6D) was confirmed to be ipsilaterally consistent with the left/right asymmetry shown in the odor columns in Fig. 2D,E. Left/right asymmetric c‐Fos expression was not observed in experiments under multi using the AONpE‐lesioned mice (Fig. 6E). In addition, a quantitative analysis indicated that the level of c‐Fos expression in both hemispheres of the olfactory tubercle in the AONpE‐lesioned mice was lower than that of an intensively c‐Fos‐labeled hemisphere in the unlesioned mice (Fig. 6C, left hemisphere; Fig. 6D, right hemisphere; Fig. 6E. *P* < 0.05 by unpaired Student’s *t*‐test. Left olfactory tubercle, *P* < 0.0001; right olfactory tubercle, *P* < 0.0001). Taken together, the coincidence of c‐Fos expression patterns under all odor exposure conditions suggests connectivity between the olfactory tubercles (Fig. 6) and the two lateral odor columns (Figs 2 and 5). In particular, the asymmetry of c‐Fos expression in the olfactory tubercles under multi was consistent with the patterns of the two lateral odor columns, probably due to synaptic linkage via the AONpE.

We quantitatively analyzed c‐Fos expression in both hemispheres of the anterior piriform cortex but did not detect asymmetric distribution of c‐Fos^+^ cells between the left and right hemispheres of the anterior piriform cortex under any of the odor exposure conditions (Fig. 7). The lowest expression of c‐Fos was observed under the single (Fig. 7A), whereas abundant c‐Fos expression was observed under the continuous and multiple pulsed conditions (Fig. 7B,C). The level of c‐Fos expression in the anterior piriform cortex was statistically the same between the continuous and multiple pulsed conditions. The AONpE‐lesioned mice under multi revealed reduced c‐Fos expression compared with normal mice under multi (Fig. 7D). In conclusion, the anterior piriform cortex did not show a consistent activity pattern compared with the odor columns, although the circuitry of the odor columns was directly connected. Unlike the odor columns and olfactory tubercle, the anterior piriform cortex did not reveal asymmetry of c‐Fos expression patterns under any of the odor exposure conditions.

## Discussion

In this study, we integrated specific OR identified mice with three different odor exposure designs and immunolabeling to elucidate the c‐Fos expression patterns in the OB and olfactory cortex depending on the odor exposure method. We also identified the distribution of the c‐Fos^+^ cell types in the odor columns and characterized the correlation of c‐Fos expression level between the odor columns and the olfactory cortex.

We designed several experimental conditions to substantiate our hypothesis according to previous studies. First, MOR23‐IRES‐tauGPF mice and lyral were utilized for our experiments. A recent study showed that the formation of two exact pairs of glomeruli by MOR23 is much more reproducible than glomeruli formed by other ORs [29]. The known MOR23 ligand lyral is narrowly tuned to the MOR23 and activates only some ORs, including the MOR23 [30, 31]. In addition, in our tile scan image of coronal sectioned OBs with c‐Fos immunolabeling, the immunoreacted columns were confirmed to be restricted to the MOR23 glomerulus, and few glomeruli were located around the MOR23 glomerulus, indicating that we had suitable tools for observing the specific interactions among the four isogenetic columns (Fig. S5). Second, we utilized awake (unanesthetized) mice in their home cage with bedding used for a few days to stimulate the odorant. Differences in olfactory activation between awake and anesthetized mice have been characterized in many studies [32, 33]. To avoid stimulation by external odors in the home cage, the experiment was carried out using the home cage from which the mice lived. Third, the duration of odor exposure was set to 90 min, as 90 min after odor stimulation results in the most abundant c‐Fos expression in the olfactory system [34]. We categorized the number of cases of odors in the environment and mimicked them in the three experimental conditions. In particular, multi, consisting of repeated sets of 1 min of odor and 4 min of fresh air, was considered the time for resensitization after adaptation of the ORs [14, 15]. The regions of interest were selected as the odor columns, the olfactory tubercle, and the anterior piriform cortex, which were directly connected by OSNs and mitral/tufted cells.

Parallel c‐Fos expression was observed between the two lateral columns and the ipsilateral olfactory tubercles under all odor exposure conditions, whereas c‐Fos expression in the left/right piriform cortex did not show parallel patterns with the ipsilateral odor columns. More importantly, in multi, one of the lateral odor columns and the ipsilateral olfactory tubercle were selectively and intensively c‐Fos labeled but less c‐Fos expression was observed in the other lateral column and contralateral olfactory tubercle via the AONpE (Fig. S6). This asymmetric c‐Fos expression was not observed in the piriform cortex under any of the odor exposure conditions. These results raise some important questions. In multi, why were the medial and one of lateral columns not selected to the intensively c‐Fos‐labeled column and why did they express limited c‐Fos regardless of communication via the AONpE? In spite of receiving inhibitory interbulbar signals from the contralateral column, what characteristics or synaptic linkage of the intensively c‐Fos‐labeled columns affect it selectivity? Studying the cell‐type population of each column and sophisticated circuitry of the medial glomeruli could be helpful when addressing these issues and to understand the selective c‐Fos expression patterns. Furthermore, molecular profiling analysis of odor columns using LCM (laser capture microdissection) combined with proteomics may provide clue to the mechanisms of lateralization.

Previous studies have reported that the AONpE is essential to sense the left/right direction of the odor source or for bilateral exchange of odorant‐specific information [9, 10, 27, 28]. Here, our data indicate that the interbulbar link through the AONpE may inhibit c‐Fos expression in one of the lateral odor columns by exchanging odor information with the contralateral odor column in a specific olfactory environment. This asymmetric c‐Fos expression pattern under multi converted to the symmetric c‐Fos expression pattern in the AONpE‐lesioned mice. A well‐known circuit of the interbulbar synaptic network is that the AONpE receives the axons of mitral/tufted cells from the ipsilateral odor column and sends neural fibers to the granule cells beneath the contralateral isogenetic column [9, 10]. As this olfactory circuit is likely to be turned on by an odorant cue, multi seems to be the most effective odor environment for interbulbar communication between odor columns than the other conditions. We hypothesized the following scenario for asymmetric c‐Fos expression in the two isogenetic lateral columns under multi: The activated lateral glomerulus transfers neural stimulation to the associated mitral/tufted cells, which, in turn, activate their second neurons in the AONpE, followed by the next targeting deep short‐axon cells in granular cell layer beneath the contralateral column. The activated deep short‐axon cells may reduce the activity of JG cells around the targeted glomerulus at the same time as its OSNs activate the odor column [35, 36]. On the other hand, the adaptation of the MOR23 is expected to occur because lyral is continuously present around the MOR23 for 90 min under the continuous [14, 15, 18]. Therefore, it is considered that the c‐Fos expression was not observed in JG cells because of low inhibitory interbulbar communication by the AONpE. Taken together, we propose that the AONpE may play a novel role suppressing c‐Fos expression in one of the lateral columns by transferring neural communication from the contralateral column in a specific olfactory environment.

The bilateral synaptic linkage via the AONpE, which conveys inhibitory information, is reminiscent of functions of local interneurons in the antennal lobe of Drosophila, the first layer interneurons of *Caenorhabditis elegans*, and the spiny neurons of *Xenopus laevis* tadpoles. These animals have been studied for their inhibitory synaptic structure or function between bilateralized olfactory systems. The local interneurons of Drosophila are connected across the interbulbar glomeruli and modulate olfactory information between the left and right hemispheres using inhibitory neurotransmitters and neuropeptides [37, 38, 39]. The olfactory system of *C. elegans* has no structure like the glomerulus but has interneurons, such as AIA and AIB, with inhibitory synaptic connections that integrate signals from bilateral primary olfactory neurons [40, 41]. In addition, the spiny neurons, which are the granule cells of *X. laevis* tadpoles, are also located in the post layer of mitral and periglomerular cells, which may modulate olfactory information from the odor columns [42, 43, 44, 45, 46]. Most animal species have two bilateral olfactory systems, but no physiological functions have been identified. If interhemispheric inhibition is functionally necessary in other species, the unilateral c‐Fos expression patterns may be expected in response to a pulsed odor input or a similar olfactory environment.

Ultimately, our results provide some clues for the existence of a distinct odor column compared to the other three columns in specific olfactory environments. The proportion of calbindin‐expressing c‐Fos^+^ cells in a lateral column under the continuous was higher than that of the other three columns (Fig. 3B,C). In addition, it was statistically equal to the proportion of calbindin‐expressing c‐Fos^+^ cells in an intensively c‐Fos‐labeled column under multi (Fig. 3B–D. unpaired Student’s *t*‐test. *P* = 0.5728, LL in Fig. 3B and intensively c‐Fos‐labeled lateral (IL) in Fig. 3D; *P* = 0.5326, RL in 3C and IL in 3D, LL; RL; IL). Comparing these two results, it is possible that one of the lateral columns could be a distinct glomerulus or column in the olfactory system. Thus, our results propose that the structure of the OB and distribution of isogenetic glomeruli is left/right symmetric but that JG cells in the odor column asymmetrically expressed c‐Fos under a pulsed odor environment. Similarly, asymmetric patterns will be identified in lateralized olfactory systems of humans or other species by pulsed odor environments in future studies.

## Materials and methods

### Mice

MOR23‐IRES‐tauGFP mice (C57/BL6J background, 8–15 weeks old, no sex consideration) were used in all experiments. All mice were housed in a room under a 12‐h light/dark cycle in a ventilated cage (M‐BTM‐C8, Innocage; Fisher Scientific, Pittsburgh, PA, USA) with odorless bedding. All experiments were followed the guidelines on care and use of laboratory animals as approved by the Daegu Gyeongbuk Institute of Science and Technology’s Institutional Animal Care and Use Committee (DGIST‐IACUC).

### Odorant exposure chamber and odor preparation

An odor exposure chamber (volume = 6 L) with two air flow meters (6 L·min^−1^) was used to apply fresh air or lyral, respectively. A disposable odor chamber was made from a home cage with used bedding. Highly dehydrated, dust purified fresh air (dew point −80 °C, filter pore sizes of 5, 3, 1, 0.01 µm, and 0.01 p.p.m., serially) was used to apply the odorant. Lyral (kindly provided by Taytonn Pte. Ltd., Singapore, or purchased from Sigma‐Aldrich, St Louis, MO, USA, 95594) was used as the odorant in this study. Pure lyral was prepared as a 10 mm stock solution in mineral oil. Before the experiment, a cotton ball (11951925; ThermoFisher Scientific, Waltham, MA, USA) was soaked with lyral solution and placed in an odor jar (volume = 225 mL). After 9 h of exposure to fresh air, the mouse was stimulated with lyral for 90 min under three different conditions. The mouse was allowed to move freely around the odor chamber.

### Immunohistochemistry

Mice were first anesthetized with 350 mg ketamine (400 mg·kg^−1^ of body weight, i.p.) and transcardially perfused with PBS (room temperature) and 4% paraformaldehyde in PBS (filtered, pH 7.4, 4 °C). Extracted brains with complete OBs were postfixed for 4 h in ice‐cold fixative solution then stored 3 days in a cryoprotectant solution (30% sucrose in PBS) at 4 °C. Cryopreserved brains with OCT compound (#4583; Scigen, Gardena, CA, USA) were coronal sliced into 14 µm for the OB and 25 µm for the brain with a cryotome (HM 550; ThermoFisher Scientific) and collected onto Superfrost‐plus microscope slides (S9441; Matsunami, Tokyo, Japan). The slides were blocked for 1 h in 4% normal horse serum (Jackson ImmunoResearch, West Grove, PA, USA) and 0.1% Triton X‐100 (T878; Sigma‐Aldrich) in PBS and were incubated with primary antibodies (1 : 200, goat anti‐c‐Fos, ab156802; Abcam, Cambridge, MA, USA; 1 : 200, rabbit anti‐c‐Fos, sc‐52; Santa Cruz Biotechnology, Santa Cruz, CA, USA; 1 : 1000, mouse anticalbindin, c9848; Sigma‐Aldrich; 1 : 10 000, rabbit anti‐TH, ab112; Abcam; 1 : 2000, and goat anticalretinin, ab1550; Millipore, Billerica, MA, USA) overnight at 4 °C. To visualize the bound primary antibodies, cy3‐conjugated anti‐goat/rabbit IgG (1 : 2000; Jackson ImmunoResearch) and cy5‐conjugated anti‐mouse/rabbit/goat IgG (1 : 2000; Jackson ImmunoResearch) diluted in 0.1% PBST were incubated for 1 h at room temperature. The sections were rinsed three times for 5 min each in PBS after incubation of the primary and secondary antibodies. Slices were preserved with mounting medium (#H‐1200; Vectashield with DAPI; Vector Laboratories, Burlingame, CA, USA) at 4 °C.

### Confocal imaging and 3D reconstruction

Fluorescent images were obtained with a laser scanning confocal microscope (LSM 700; Zeiss, Oberkochen, Germany). A complete series of slices containing the entire GFP structure was captured for each odor column image. All images from the OB were captured with a 20× lens and were 212.35 × 212.35 µm in area, which was sufficient to accommodate the odor column. The 14‐µm‐thick OB tissues were z‐stack and captured in four planes for the statistical analysis or captured in 14 planes to prepare 3D movie clips. Every 100 µm from bregma 2 mm to bregma 0 mm was tile scan imaged for the images of the olfactory tubercle and anterior piriform cortex. All images were captured in blue for nuclei, green for the GFP^+^ odor columns, red for c‐Fos, and cyan for the cell‐type markers. Cy5, the far‐red wavelength dye, converted to cyan color. Neurolucida360 (MBF Bioscience, Williston, VT, USA) was used for 3D reconstruction from the series of single‐plane images of the GFP^+^ odor column.

### Quantitative analysis

All quantitatively analyzed images were obtained from 5–20 mice in each experimental condition. Four odor columns, the olfactory tubercle, and the anterior piriform cortex were analyzed without capture failure. A total of 1985 image planes were evaluated for the quantitative analysis. Each odor column was sectioned and analyzed into 5–9 divided z‐stack image files, and the counted values of each images were summed up. Every fourth section from the serial sections of the target area (from bregma 2 mm to bregma 0 mm) were quantitatively analyzed and summed up for the olfactory tubercle and the anterior piriform cortex. The quantitative analysis of the JG cells of the MOR23‐targeted glomeruli (MOR23 glomeruli) was performed according to well‐established counting methods [47, 48]. First, GFP‐expressing neuropils surrounded by DAPI‐stained JG cells were defined as the MOR23 glomeruli. Second, the region of interest was limited to less than four nuclear widths from the outer boundary of the MOR23 targeted glomerulus. Third, only the red channel for c‐Fos in the nucleus or the far‐red channel for the cell markers in the cytoplasm was used for the analysis (Fig. S2).

### Statistical analysis

All data are shown as mean ± SEM. Differences were detected using one‐way analysis of variance (ANOVA) or the unpaired Student’s *t*‐test to compare repeated measurements and their respective control values, using graphpad prism 5 software (GraphPad Software, La Jolla, CA, USA).

### Brain lesions and ibotenic acid injections

Mice were immobilized in a stereotactic frame (# 51730; Stoelting Co., Wood Dale, IL, USA) after anesthesia with 350 mg ketamine (400 mg·kg^−1^ of body weight, i.p.). A 1 µL aliquot of 0.5% IBA (I2765; Sigma‐Aldrich) in PBS was pressure‐injected into the target area to produce lesions in the left and right sides of the AONpE. Infusion speed was 0.2 µL·min^−1^. The injection site was bregma −3.25 mm, 1.2 mm left or right from the longitudinal fissure, at a depth of 1.9 mm in the skull. The injected region followed well‐established references [10, 29]. The volumes of the lesion regions in the AONpE were at least five times larger than the established volume of the synaptic area by targeting external tufted cells (Fig. S4). The lesion solution was mixed with 0.2 µL biotinylated dextran amine (SP‐1140; Vector Laboratories) to visualize the lesion sites. After the infusion, the cannula remained in place for 5 min. IBA was administered 14 days before the odorant‐stimulating experiment was conducted.

### Preparation of the MOR23‐expressing olfactory sensory neuron

MOR23‐IRES‐tauGFP mice were killed by cervical dislocation. The head was opened with a razor blade and the septal olfactory epithelium was dissected and maintained in 1 mL of ice‐cold aCSF supplemented with 10 mm glucose (oxygenated with 95% O_2_). The extracted tissue was transferred into calcium‐free aCSF supplemented with 1 mg·mL^−1^ papain (P3250; Sigma‐Aldrich) and 10 unit per mL DNase (11284932001; Roche, Basel, Swiss). After incubation with two enzymes for 15 min at 37 °C in 5% CO_2_, the tissue was gently rinsed two times with 1 mL of oxygenated aCSF, chopped with a disposable scalpel, and gently triturated with a glass pipette in 1 mL of aCSF. The 1 mL of cell suspension was filtered through a 40‐μm cell strainer (352340; BD Biosciences, San Jose, CA, USA) and stored 1‐mL tube at 4 °C.

### Calcium imaging with the lyral

The filtered cells were incubated for 20 min at 37 °C with 10 μm Rhod‐3, AM (R10145, ThermoFisher Scientific) and 0.04% Pluronic‐F127 (P2433; Sigma‐Aldrich) contained aCSF in a bath chamber (RC‐42LP; Warner Instruments, Hamden, CT, USA). After Rhod‐3, AM incubation, chamber was placed on a stage of inverted microscope (NIKON ECLIPSE Ti, Tokyo, Japan) then washing with aCSF (room temperature) for 5 min before lyral application. The aCSF and lyral were perfused through tubing with a peristaltic pump at a flow rate of 2 mL·min^−1^. 100 μm lyral diluted with 0.1% DMSO in oxygenated aCSF was applied for 15 s. After treatment of lyral, mixture solution of 100 μm IBMX and 10 μm forskolin was applied for confirmation of neuronal viability. The intracellular Ca^2+^ level was measured by 610 nm, emission of Rhod‐3, AM fluorescence ratio (excitation, 545 nm). Emitted fluorescence was recorded every 2 s using CCD camera (Andor Technology, Belfast, UK). Analysis for intensity of fluorescence was carried out using MetaFluor (Molecular Devices, Sunnyvale, CA, USA). All pseudocolor images were converted from fractional fluorescence change (Δ*F*/*F*, Δ[Ca^2+^]_i_), corresponding to changes in intracellular calcium ion concentration.

## Conflict of interest

The authors declare no conflict of interest.

## Author contributions

YJ designed and performed the experiments. YJ and NL wrote the manuscript and analyzed the data. All authors participated in the composition of the manuscript.

## Supporting information


**Fig. S1** **.** Confirmation of lyral specificity to the MOR23. (A, B) Intracellular calcium response in GFP expressing OSNs was evoked by lyral (100 μm). (A) Peak of intracellular calcium ([Ca2+]i) was analyzed by drawing a region of interest around responding OSNs. (B) Intracellular calcium images in each time points from a to e was presented by converting 610nm fluorescent intensity (Emission wave length of Rhod‐3, AM). (C) Expression of c‐Fos in mitral/tufted cells located beneath the MOR23 glomerulus by exposure of lyral. Scale bar, 100 μm.
**Fig. S2** **.** Representative 4‐colored z‐stack images of the odor column. Serial image‐set reveals calretinin expressing c‐Fos + cells of a left lateral odor column under the continuous condition. Panels from left to right are anterior to posterior sections of the odor column. c‐Fos was labeled with red (top and bottom panels) and calretinin was visualized with cyan (middle panels) or green (bottom panels). Yellow cells in the bottom panel were counted as calretinin expressing c‐Fos + cells for the quantitative analysis. Scale bar, 100 μm.
**Fig. S3** **.** Three antibodies (TH, calbindin, and calretinin) for JG cells were immunolabeled on the OB near the MOR23 glomerulus area. (A), Visualized TH with green; (B), visualized calbindin with red; (C), visualized calretinin with yellow; (D), merged image. The three kind of antibodies labeled different cells within the cell bodies. Scale bar, 50 μm.
**Fig. S4** **.** AONpE‐lesioned area by ibotenic acid injection. Ibotenic acid was mixed with biotinylated dextran amine to visualize the lesioned region. Neurons in the center of the injection site (inside dashed area) were completely lesioned. The thickness of the lesioned region was at least 360 μm (40 μm thickness brain tissue × 9 analyzed sections). Scale bar, 0.5 mm.
**Fig. S5** **.** c‐Fos labeled cells were abundantly located around the MOR23 glomerulus in the OB under the single pulsed condition in wide scan image of coronal sectioned tissue. Scale bar, 100 μm.
**Fig. S6**. Representative activation pattern of the olfactory‐related regions in the brain under the multiple pulsed condition. Abundant c‐Fos immunoreactivity was observed in the left lateral odor column and olfactory tubercle in the left hemisphere but the other three odor columns and the olfactory tubercle in the right hemisphere were not. Piriform cortex revealed symmetric c‐Fos immunoreactivity between the left and right hemispheres. Bregma coordinates are shown on the right side of each figure. Scale bar, 100 μm.
**Table S1** **.** Quantitative analysis of calbindin expressing c‐Fos^+^ cells in the four odor columns depending on the three different odor exposure conditions (related to Fig. 3)
**Table S2** **.** Quantitative analysis of TH expressing c‐Fos^+^ cells in the four odor columns depending on the three different odor exposure conditions (related to Fig. 4)
**Table S3** **.** Quantitative analysis of calretinin expressing c‐Fos^+^ cells in the four odor columns depending on the three different odor exposure conditions (related to Fig. 4)
**Table S4** **.** Cell counting for c‐Fos expression patterns in the odor columns following the multiple pulsed condition after injecting IBA or PBS (related to Fig. 5)
**Table S5** **.** Cell counting for c‐Fos expression in the odor columns following the multiple pulsed condition in single‐side AONpE‐lesioned mice
**Table S6** **.** Cell counting of c‐Fos expression in the olfactory tubercle depending on the three different odor exposure conditions and multiple pulsed condition after injecting IBA (related to Fig. 6)
**Table S7** **.** Cell counting of c‐Fos expression in the piriform cortex depending on the three different odor exposure conditions and multiple pulsed condition after injecting IBA (related to Fig. 7)
**Video S1** **.** Activated left lateral column.
**Video S2** **.** Left medial column.
**Video S3** **.** Right medial column.
**Video S4** **.** Right lateral column.
