# Supplementary material for: Interhemispheric asymmetry of c‐Fos expression in glomeruli and the olfactory tubercle following repeated odor stimulation
Source: FEBS Open Bio. 2020 Apr 13;10(5):912–26. doi: 10.1002/2211-5463.12851 (PMC7193154; doi:10.1002/2211-5463.12851)
Supplement: Supplementary file 1 — Fig. S1 . Confirmation of lyral specificity to the MOR23. (A, B) Intracellular calcium response in GFP expressing OSNs was evoked by lyral (100 μm). (A) Peak of intracellular calcium ([Ca2+]i) was analyzed by drawing a region of interest around responding OSNs. (B) Intracellular calcium images in each time points from a to e was presented by converting 610nm fluorescent intensity (Emission wave length of Rhod‐3, AM). (C) Expression of c‐Fos in mitral/tufted cells located beneath the MOR23 glomerulus by exposure of lyral. Scale bar, 100 μm. Fig. S2 . Representative 4‐colored z‐stack images of the odor column. Serial image‐set reveals calretinin expressing c‐Fos + cells of a left lateral odor column under the continuous condition. Panels from left to right are anterior to posterior sections of the odor column. c‐Fos was labeled with red (top and bottom panels) and calretinin was visualized with cyan (middle panels) or green (bottom panels). Yellow cells in the bottom panel were counted as calretinin expressing c‐Fos + cells for the quantitative analysis. Scale bar, 100 μm. Fig. S3 . Three antibodies (TH, calbindin, and calretinin) for JG cells were immunolabeled on the OB near the MOR23 glomerulus area. (A), Visualized TH with green; (B), visualized calbindin with red; (C), visualized calretinin with yellow; (D), merged image. The three kind of antibodies labeled different cells within the cell bodies. Scale bar, 50 μm. Fig. S4 . AONpE‐lesioned area by ibotenic acid injection. Ibotenic acid was mixed with biotinylated dextran amine to visualize the lesioned region. Neurons in the center of the injection site (inside dashed area) were completely lesioned. The thickness of the lesioned region was at least 360 μm (40 μm thickness brain tissue × 9 analyzed sections). Scale bar, 0.5 mm. Fig. S5 . c‐Fos labeled cells were abundantly located around the MOR23 glomerulus in the OB under the single pulsed condition in wide scan image of coronal sectioned tissue. Scale bar, 100 [file FEB4-10-912-s001.pdf]

Supporting Information

Interhemispheric asymmetry of c-Fos expression in glomeruli and the olfactory tubercle following repeated odor stimulation

YoonGyu Jae[1]*, NaHye Lee[1], Dae won Moon[2], JaeHyung Koo[2,3]

[1]Department of Brain and Cognitive Sciences, DGIST, Dalseong, Daegu, 42988 Republic of Korea; [2]Department of New Biology, DGIST, Dalseong, Daegu, 42988 Republic of Korea; [3]Center for Bio-Convergence Spin System, DGIST, Dalseong, Daegu, 42988 Republic of Korea

Supplementary Figures

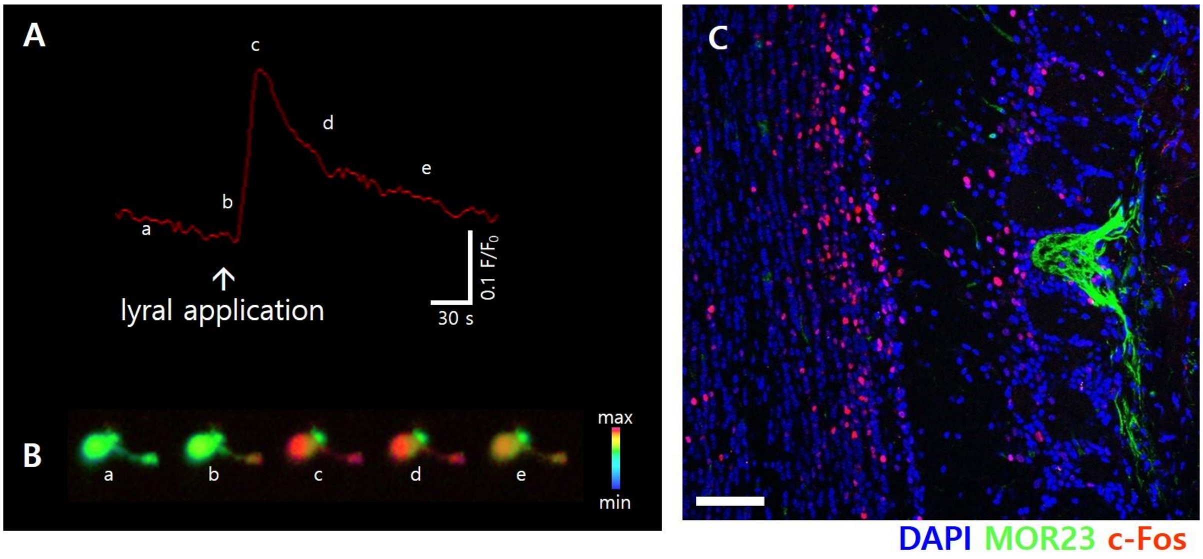

Supplementary Figure 1. Confirmation of lyral specificity to the MOR23. (A, B) Intracellular calcium response in GFP expressing olfactory sensory neuron was evoked by lyral (100μM). (A) Peak of intracellular calcium ($[Ca^{2+}]_i$) was analyzed by drawing a region of interest around responding olfactory sensory neuron. (B) Intracellular calcium images in each time points from a to e was presented by converting 610nm fluorescent intensity (Emission wave length of Rhod-3, AM). (C) Expression of c-Fos in mitral/tufted cells located beneath the MOR23 glomerulus by exposure of lyral. Scale bar, 100 μm.

(Next page)

Supplementary Figure 2. Representative 4-colored z-stack images of the odor column. Serial image-set reveals calretinin expressing c-Fos^+ cells of a left lateral odor column under the continuous condition. Panels from left to right are anterior to posterior sections of the odor column. c-Fos was labeled with red (top and bottom panels) and calretinin was visualized with cyan (middle panels) or green (bottom panels). Yellow cells in the bottom panel were counted as calretinin expressing c-Fos^+ cells for the quantitative analysis. Scale bar, 100 μm.

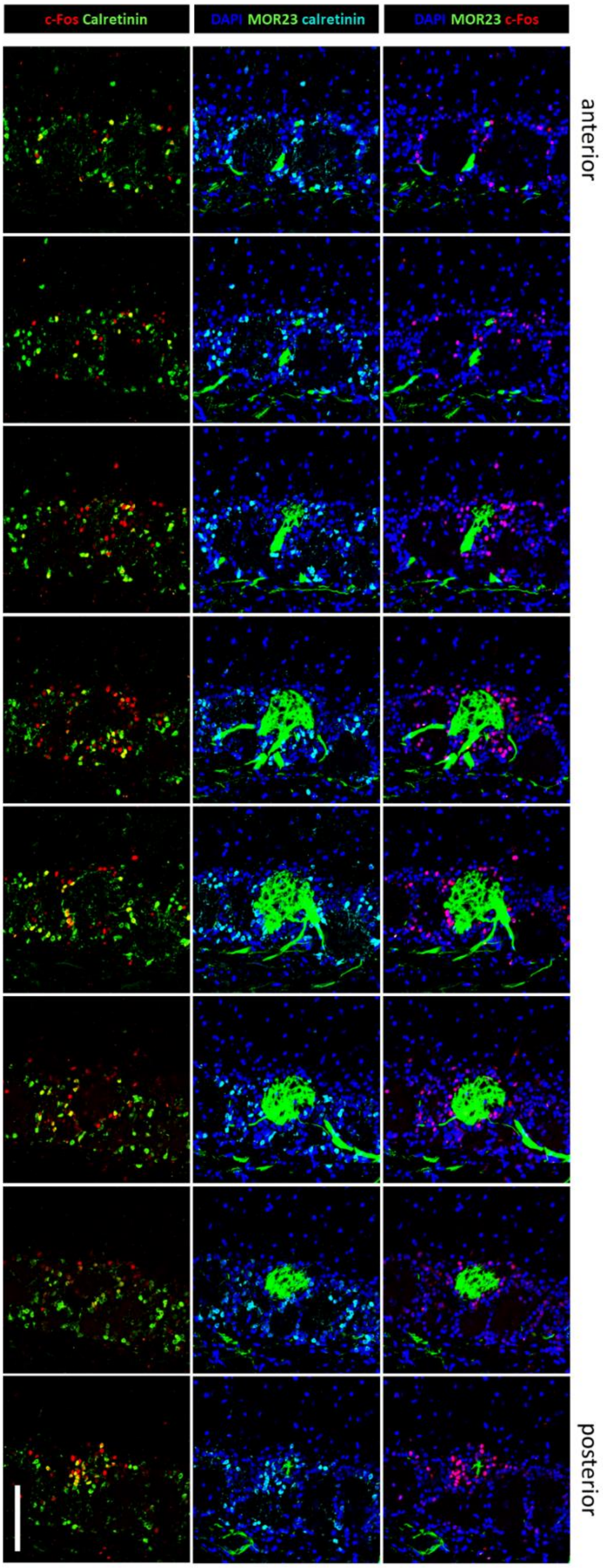

c-Fos Calretinin
DAPI MOR23 calretinin
DAPI MOR23 c-Fos
anterior
posterior

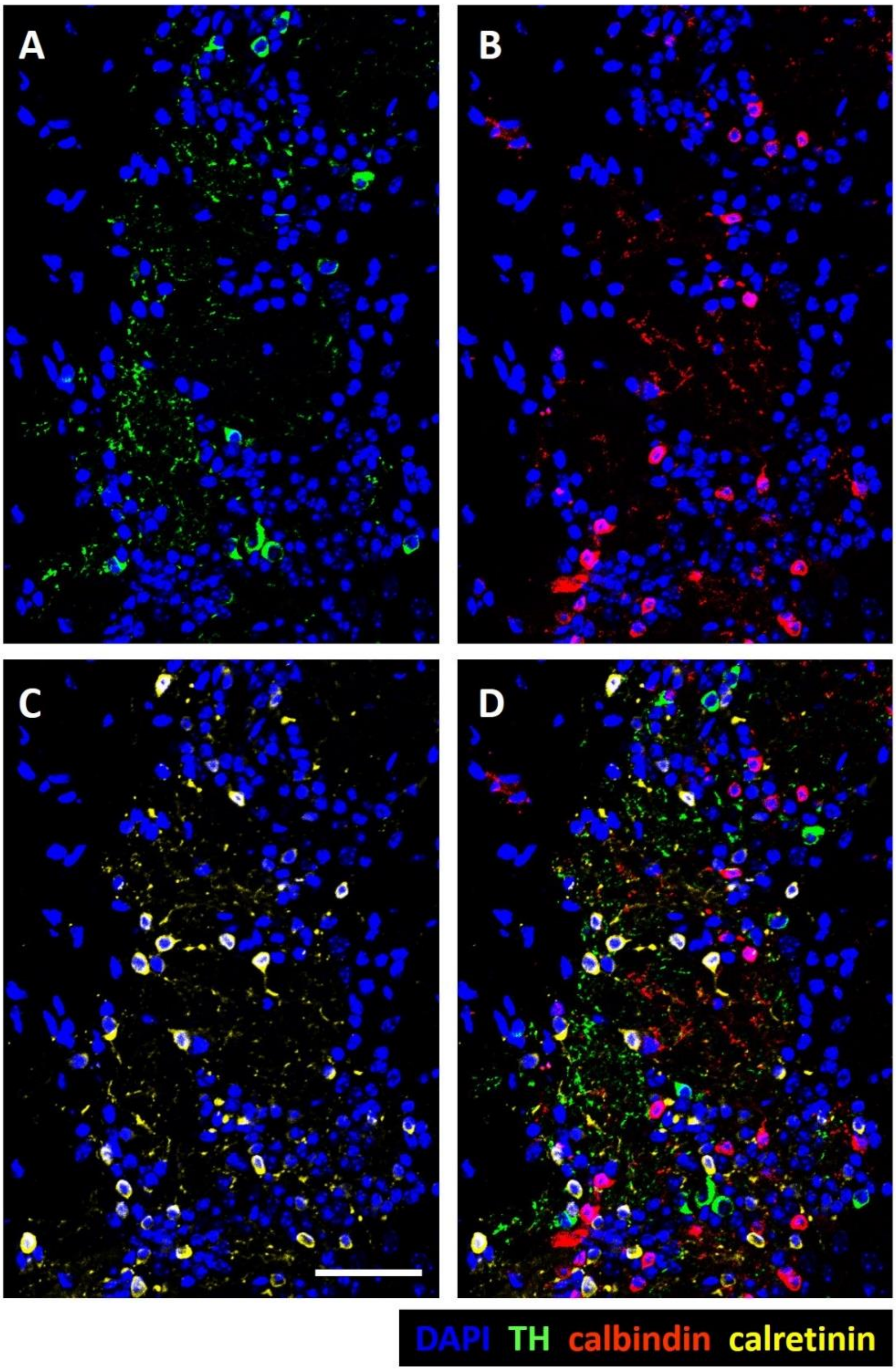

Supplementary Figure 3. Three antibodies (TH, calbindin, and calretinin) for JG cells were immunolabeled on the OB near the MOR23 glomerulus area. **(A)**, Visualized TH with green; **(B)**, visualized calbindin with red; **(C)**, visualized calretinin with yellow; **(D)**, merged image. The three kind of antibodies labeled different cells within the cell bodies. Scale bar, 50 µm.

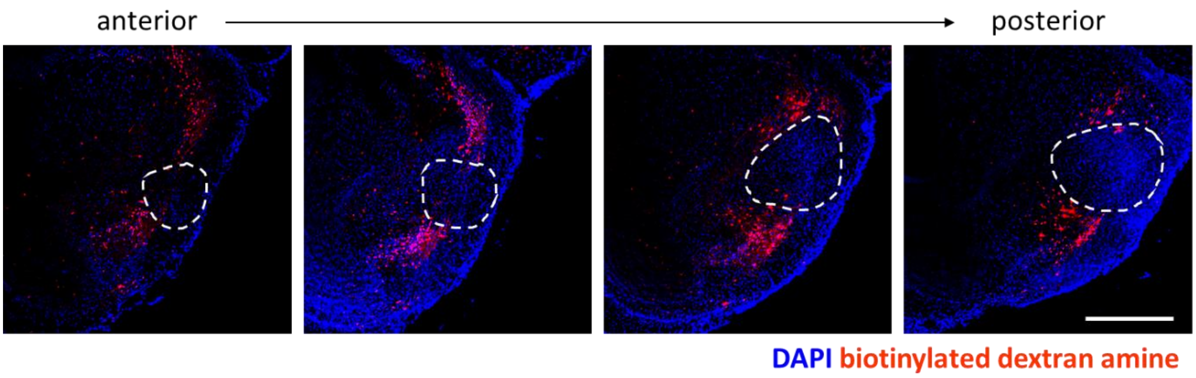

Supplementary Figure 4. AONpE-lesioned area by ibotenic acid injection. Ibotenic acid was mixed with biotinylated dextran amine to visualize the lesioned region. Neurons in the center of the injection site (inside dashed area) were completely lesioned. The thickness of the lesioned region was at least 360 µm (40 µm thickness brain tissue × 9 analyzed sections). Scale bar, 0.5 mm.

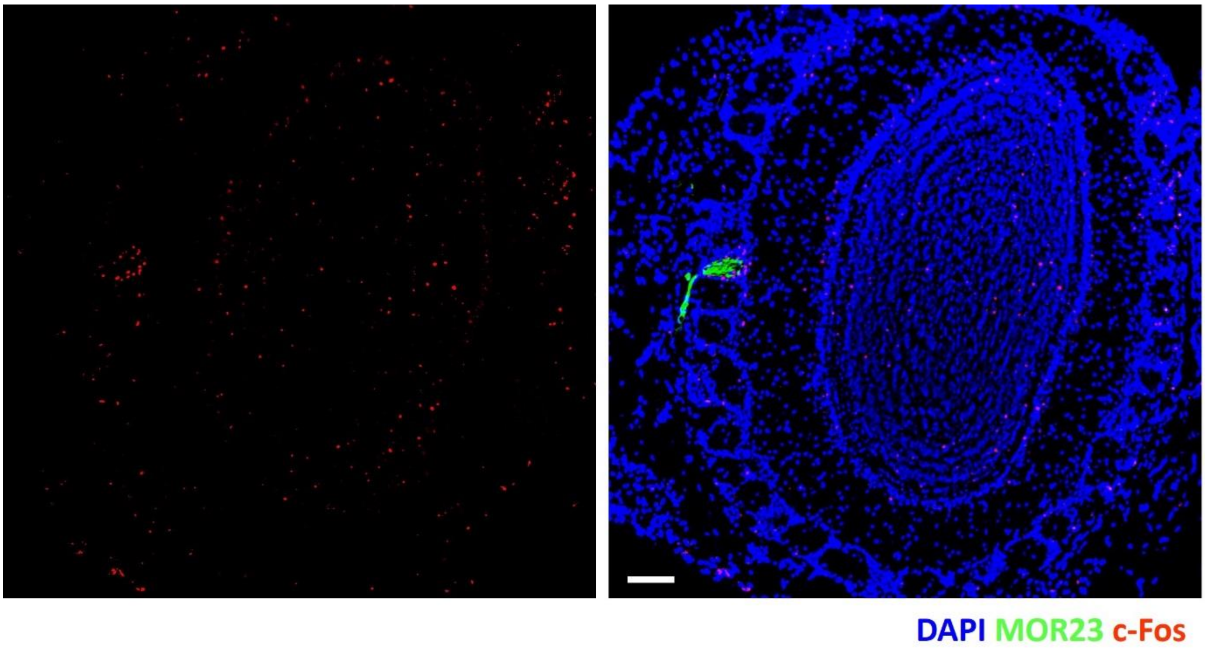

Supplementary Figure 5. c-Fos labeled cells were abundantly located around the MOR23 glomerulus in the OB under the single pulsed condition in wide scan image of coronal sectioned tissue. Scale bar, 100 µm.

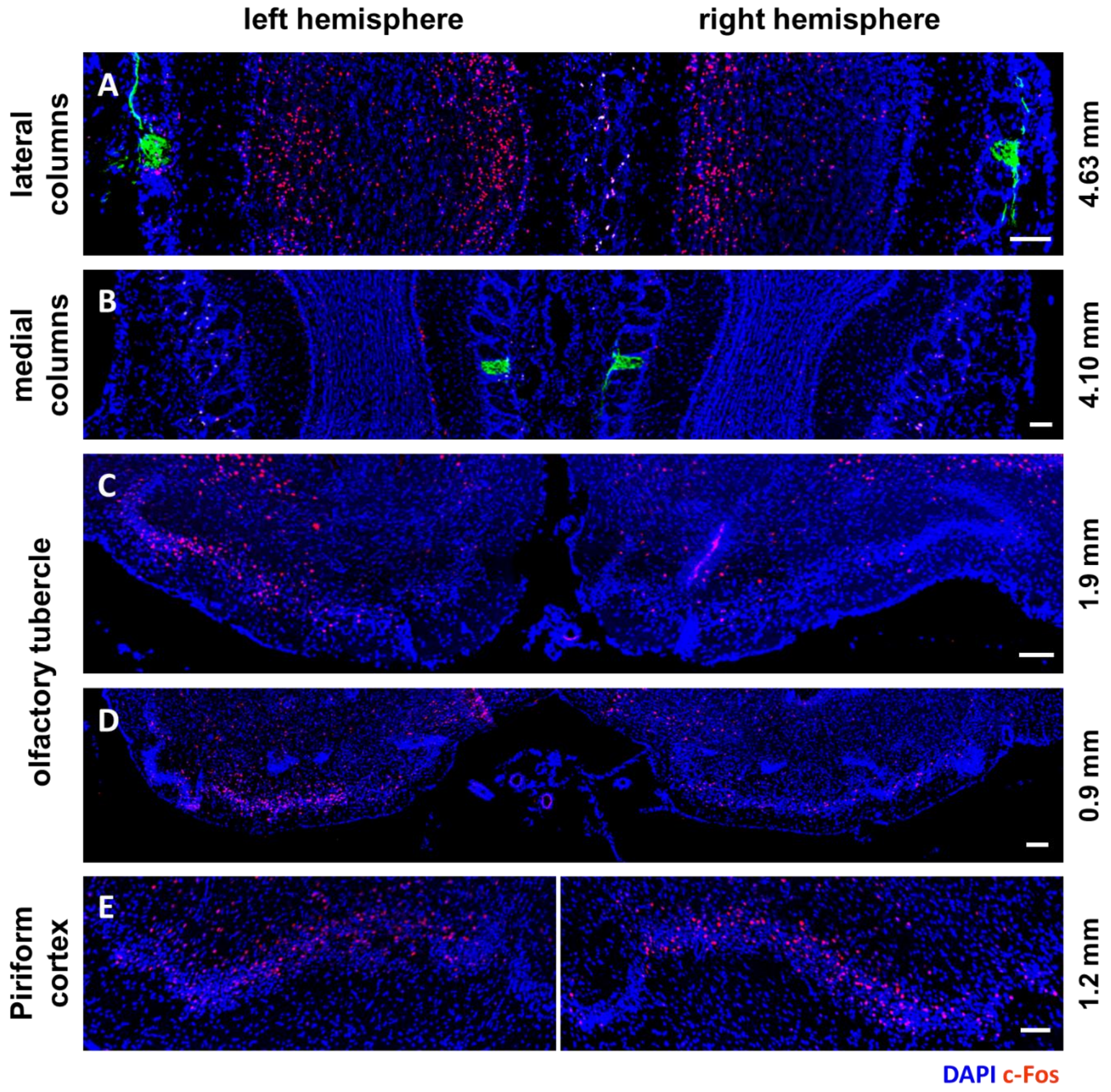

Supplementary Figure 6. Representative activation pattern of the olfactory-related regions in the brain under the multiple pulsed condition. Abundant c-Fos immunoreactivity was observed in the left lateral odor column and olfactory tubercle in the left hemisphere but the other three odor columns and the olfactory tubercle in the right hemisphere were not. Piriform cortex revealed symmetric c-Fos immunoreactivity between the left and right hemispheres. Bregma coordinates are shown on the right side of each figure. Scale bar, 100 μm.

Supplementary Tables

Supplementary Table 1. Quantitative analysis of calbindin expressing c-Fos^+ cells in the four odor columns depending on the three different odor exposure conditions (related to Figure 3)

Supplementary Table 2. Quantitative analysis of TH expressing c-Fos^+ cells in the four odor columns depending on the three different odor exposure conditions (related to Figure 4)

Supplementary Table 3. Quantitative analysis of calretinin expressing c-Fos^+ cells in the four odor columns depending on the three different odor exposure conditions (related to Figure 4)

Supplementary Table 4. Cell counting for c-Fos expression patterns in the odor columns following the multiple pulsed condition after injecting IBA or PBS (related to Figure 5)

Supplementary Table 5. Cell counting for c-Fos expression in the odor columns following the multiple pulsed condition in single-side AONpE-lesioned mice

Supplementary Table 6. Cell counting of c-Fos expression in the olfactory tubercle depending on the three different odor exposure conditions and multiple pulsed condition after injecting IBA (related to Figure 6)

Supplementary Table 7. Cell counting of c-Fos expression in the piriform cortex depending on the three different odor exposure conditions and multiple pulsed condition after injecting IBA (related to Figure 7)

Supplementary Table 1. Quantitative analysis of calbindin expressing c-Fos+ cells in the four odor columns depending on the three different odor exposure conditions **(related to Figure 3)**

	# of c-Fos+ cells				# of calbindin expressing c-Fos+ cells				proportion of calbindin expressing c-Fos+ cells			
	LL	LM	RM	RL	LL	LM	RM	RL	LL	LM	RM	RL
single	82	57	82	60	4	1	6	2	4.88	1.75	7.32	3.33
	80	92	49	89	2	3	1	3	2.50	3.26	2.04	3.37
	73	80	69	71	4	4	6	3	5.48	5.00	8.70	4.23
	45	35	38	82	3	2	4	2	6.67	5.71	10.53	2.44
	28	55	44	59	1	3	3	2	3.57	5.45	6.82	3.39
conti	113	83	97	102	24	4	3	8	21.24	4.82	3.09	7.84
	100	103	79	82	25	4	5	8	25.00	3.88	6.33	9.76
	94	26	40	95	24	2	6	4	25.53	7.69	15.00	4.21
	81	75	71	99	19	4	5	5	23.46	5.33	7.04	5.05
	82	87	74	84	21	8	7	4	25.61	9.20	9.46	4.76
	81	65	83	83	19	4	8	6	23.46	6.15	9.64	7.23
	45	53	45	62	17	5	7	10	37.78	9.43	15.56	16.13
	117	101	94	123	4	5	3	25	3.42	4.95	3.19	20.33
	112	94	84	134	7	6	4	26	6.25	6.38	4.76	19.40
	84	92	102	92	6	5	4	21	7.14	5.43	3.92	22.83
	102	81	91	98	6	5	6	26	5.88	6.17	6.59	26.53
	100	90	81	104	2	3	6	30	2.00	3.33	7.41	28.85
	42	47	40	79	7	3	6	34	16.67	6.38	15.00	43.04
multi	54	6	9	7	12	0	2	1	22.22	0.00	22.22	14.29
	82	0	0	3	21	0	0	0	25.61	0.00	0.00	0.00
	92	0	0	9	17	0	0	1	18.48	0.00	0.00	11.11
	9	4	1	141	2	1	0	29	22.22	25.00	0.00	20.57
	5	3	5	125	1	0	0	27	20.00	0.00	0.00	21.60
	7	4	1	98	1	0	0	31	14.29	0.00	0.00	31.63
	13	2	1	93	2	1	0	37	15.38	50.00	0.00	39.78
	1	0	0	116	0	0	0	12	0.00	0.00	0.00	10.34

Supplementary Table 2. Quantitative analysis of TH expressing c-Fos^{+} cells in the four odor columns depending on the three different odor exposure conditions **(related to Figure 4)**

	# of c-Fos^{+} cells				# of TH expressing c-Fos^{+} cells				proportion of TH expressing c-Fos^{+} cells			
	LL	LM	RM	RL	LL	LM	RM	RL	LL	LM	RM	RL
single	66	20	17	57	13	3	2	5	19.70	15.00	11.76	8.77
	17	16	22	49	2	4	3	7	11.76	25.00	13.64	14.29
	45	54	36	46	8	9	7	5	17.78	16.67	19.44	10.87
	52	42	57	15	5	12	9	2	9.62	28.57	15.79	13.33
	33	42	59	47	6	3	4	5	18.18	7.14	6.78	10.64
conti	94	84	67	92	17	11	9	16	18.09	13.10	13.43	17.39
	84	94	80	138	18	11	8	31	21.43	11.70	10.00	22.46
	92	106	90	112	10	11	8	17	10.87	10.38	8.89	15.18
	102	66	104	128	19	12	15	13	18.63	18.18	14.42	10.16
	84	92	51	92	7	5	8	12	8.33	5.43	15.69	13.04
	90	80	74	84	5	7	10	23	5.56	8.75	13.51	27.38
multi	123	5	1	2	15	0	0	0	12.20	0.00	0.00	0.00
	93	2	1	4	9	0	0	0	9.68	0.00	0.00	0.00
	99	4	5	10	15	0	1	1	15.15	0.00	20.00	10.00
	82	0	0	4	20	0	0	0	24.39	0.00	0.00	0.00
	9	6	3	106	0	1	0	18	0.00	16.67	0.00	16.98
	5	2	7	129	1	0	1	20	20.00	0.00	14.29	15.50
	8	0	3	132	0	0	0	28	0.00	0.00	0.00	21.21
	1	0	0	73	0	0	0	8	0.00	0.00	0.00	10.96

Supplementary Table 3. Quantitative analysis of calretinin expressing c-Fos$^+$ cells in the four odor columns depending on the three different odor exposure conditions **(related to Figure 4)**

	# of c-Fos$^+$ cells				# of calretinin expressing c-Fos$^+$ cells				proportion of calretinin expressing c-Fos$^+$ cells			
	LL	LM	RM	RL	LL	LM	RM	RL	LL	LM	RM	RL
single	50	23	14	36	6	3	1	3	12.00	13.04	7.14	8.33
	58	12	32	90	8	2	4	12	13.79	16.67	12.50	13.33
	114	39	70	94	18	7	17	18	15.79	17.95	24.29	19.15
	75	32	37	97	9	7	3	12	12.00	21.88	8.11	12.37
	37	54	48	50	5	9	6	6	13.51	16.67	12.50	12.00
conti	116	92	77	71	67	32	24	18	57.76	34.78	31.17	25.35
	102	74	97	98	26	31	24	32	25.49	41.89	24.74	32.65
	131	78	99	64	42	21	25	30	32.06	26.92	25.25	46.88
	132	70	40	144	52	27	21	65	39.39	38.57	52.50	45.14
	106	100	98	124	63	31	52	39	59.43	31.00	53.06	31.45
multi	100	7	8	21	30	1	1	2	30.00	14.29	12.50	9.52
	43	0	3	2	27	0	1	0	62.79	0.00	33.33	0.00
	132	0	4	1	61	0	1	0	46.21	0.00	25.00	0.00
	83	3	1	4	40	1	0	1	48.19	33.33	0.00	25.00
	7	4	1	112	1	0	0	24	14.29	0.00	0.00	21.43
	8	0	3	132	0	0	0	35	0.00	0.00	0.00	26.52
	1	2	3	97	0	0	1	19	0.00	0.00	33.33	19.59
	6	3	2	80	0	0	0	19	0.00	0.00	0.00	23.75

Supplementary Table 4. Cell counting for c-Fos expression patterns in the odor columns following the multiple pulsed condition after injecting IBA or PBS **(related to Figure 5)**

	# of c-Fos$^+$ cells			
	LL	LM	RM	RL
IBA + multi	100	1	1	102
	86	3	4	93
	97	1	2	138
	111	0	1	106
	121	0	8	82
PBS+ multi	81	3	7	10
	75	2	10	2
	131	6	3	4
	119	4	8	6
	91	10	5	13
	100	6	4	12
	78	7	3	9
	12	8	6	135
	2	2	4	100
	14	6	3	74
	7	4	2	117
	9	1	2	93
	12	6	6	120

Supplementary Table 5. Cell counting for c-Fos expression in the odor columns following the multiple pulsed condition in single-side AONpE-lesioned mice

		# of c-Fos$^+$ cells			
		LL	LM	RM	RL
IBA+ multi	left AONpE lesioned	89	7	4	3
		71	12	8	12
		13	91	2	111
		4	3	87	12
		8	79	93	9
		9	5	9	132
		8	5	98	129
		77	3	2	92
	right AONpE lesioned	100	6	8	18
		82	6	93	20
		93	5	71	21
		9	69	89	5
		105	9	7	99
		132	3	92	129

Supplementary Table 6. Cell counting of c-Fos expression in the olfactory tubercle depending on the three different odor exposure conditions and multiple pulsed condition after injecting IBA **(related to Figure 6)**

	olfactory tubercle	
	left	right
fresh air	31	41
	25	17
	19	25
	11	20
	25	31
single	350	368
	495	369
	442	331
	401	345
	375	488
conti	1257	1349
	1106	1342
	1116	1141
	1384	1302
	1246	1277
multi	1362	880
	1238	585
	1182	502
	1325	532
	1289	499
	1452	711
	1132	575
	1469	618
	1019	578
	1523	753
	1399	778
	972	1398
	375	1306
	376	1226
	524	1311
	489	1276
	739	1375
	756	1299
	598	1355
	438	1262
	738	1473
	734	1212
	443	1742
	900	1681
IBA+ multi	920	866
	991	894
	770	715
	880	867
	869	998

Supplementary Table 7. Cell counting of c-Fos expression in the piriform cortex depending on the three different odor exposure conditions and multiple pulsed condition after injecting IBA **(related to Figure 7)**

	piriform cortex	
	left	right
fresh air	46	41
	42	59
	51	52
	39	33
	47	45
single	1508	1470
	1557	1502
	1371	1476
	1435	1384
	1497	1348
conti	2504	2399
	2901	2677
	2646	2593
	2314	2981
	2655	2711
	2872	2414
multi	2350	2603
	2239	2717
	2619	2437
	2951	2261
	2847	2854
	2298	2678
	2846	2473
IBA+ multi	1995	1857
	2170	2014
	1883	1684
	1945	1829
	1765	1694
	2091	1720

Supplementary Movies Representative c-Fos expression pattern of two pairs of odor columns under multiple pulsed condition. One of lateral odor columns (left lateral column) was intensively c-Fos immunolabeled, but other three odor columns poorly immunoreacted.

Supplementary Movie 1. Activated left lateral column

Supplementary Movie 2. Left medial column

Supplementary Movie 3. Right medial column

Supplementary Movie 4. Right lateral column
